# Data set on the bioprecipitation of sulfate and trivalent arsenic by acidophilic non-traditional sulfur reducing bacteria

**DOI:** 10.1016/j.dib.2017.12.064

**Published:** 2018-01-02

**Authors:** Letícia Paiva de Matos, Patrícia Freitas Costa, Mariana Moreira, Paula Cristine Silva Gomes, Silvana de Queiroz Silva, Leandro Vinícius Alves Gurgel, Mônica Cristina Teixeira

**Affiliations:** Federal University of Ouro Preto, Campus Universitário Morro do Cruzeiro, s/n°, Bauxita, 35400-000 Ouro Preto, Minas Gerais, Brazil

**Keywords:** Arsenite, Sulfate reduction, Bioremediation, Immobilized cells, Acid pH

## Abstract

Data presented here are related to the original paper “Simultaneous removal of sulfate and arsenic using immobilized non-traditional sulfate reducing bacteria (SRB) mixed culture and alternative low-cost carbon sources” published by same authors (Matos et al., 2018) [1]. The data set here presented aims to facilitate this paper comprehension by giving readers some additional information. Data set includes a brief description of experimental conditions and the results obtained during both batch and semi-continuous reactors experiments. Data confirmed arsenic and sulfate were simultaneously removed under acidic pH by using a biological treatment based on the activity of a non-traditional sulfur reducing bacteria consortium. This microbial consortium was able to utilize glycerol, powdered chicken feathers as carbon donors, and proved to be resistant to arsenite up to 8.0 mg L^−^^1^. Data related to sulfate and arsenic removal efficiencies, residual arsenite and sulfate contents, pH and Eh measurements obtained under different experimental conditions were depicted in graphical format.

Refers to https://doi.org/10.1016/j.cej.2017.11.035

**Specifications Table**

**Table:** 

Subject area	*Chemistry, Biology, Engineering*
More specific subject area	*Biotechnology processes, Bioremediation.*
Type of data	*Table, image, graph, figure*
How data was acquired	*pH and Eh measurements: digital potentiostat with a combined platinum electrode (Digimed, DM-22).*
	*Residual sulfate concentration: turbidimetric method*[2].
	*Total arsenic content: determined by inductively coupled plasma optical emission spectroscopy (ICP-OES) (Varian, 725-ES).*
Data format	*Analyzed. Averaged data*
Experimental factors	*Brief description of any pretreatment of samples*
Experimental features	*Culture media was prepared as previously described*[1], [3] and *incubated in an oven (Fanem, model A-LT). Samples were centrifuged (10,000 rpm, 15 min, Fiberlite F155-8×50cy, Thermo, Multifuge X1R) and filtered (0.45 μm acetate cellulose membrane - Sartorius-Stedium) and acidified with nitric acid (50 μL) before residual As(III) measurements.*
Data source location	*Ouro Preto, Brazil*
Data accessibility	*All data are included in this document.*
Related research article	L. P. Matos, P. F. Costa, M. Moreira, P. C. S. Gomes, S. Q. Silva, L. V. A. Gurgel, M. C. Teixeira, Simultaneous removal of sulfate and arsenic using immobilized non-traditional SRB mixed culture and alternative low-cost carbon sources, Chemical Engineering Journal, 334 (2018), 1630–1641.

**Value of the Data**

• Different experimental conditions were compared. Free and immobilized bacterial cells were used. Different organic electron donors were tested including some low cost waste material.
• Data compare results obtained under batch and semi-continuous experimental conditions.
• Semi-continuous experiments were carried out for a long time. Data were collected for more than 150 days.
• Arsenite (bio)precipitation by sulfate reducing microorganisms was achieved under acidic pH.

## 1 Data

Data described simultaneous SO_4_^2−^ and As(III) removal obtained by using a non-traditional SRB microbial consortium previously adapted to the growth under acidic pH using Glycerol and PCF as electron donors. The main bacterial species identified are: *Pantoea agglomerans*, *Enterobacter* sp., *Citrobacter* sp., *Cupriavidus metallidurans*, *Ralstonia* sp. and *Burkholderia cepacia*. Arsenic and sulfate are commonly found as contaminants in industrial effluents from mining and metallurgical industries.

Arsenic and sulfate removal were obtained under batch and semi-continuous culture conditions. Semi-continuous up-flow reactors were constructed and operated for more than 150 days to prove their efficiency. At the final, effluent pH was neutralized and, depending on the operational conditions, SO_4_^2^^−^ and As(III) ions were removed with 74.8% and 80% efficiency, respectively.

### 1.1 Batch experiments

**Fig. 1:**
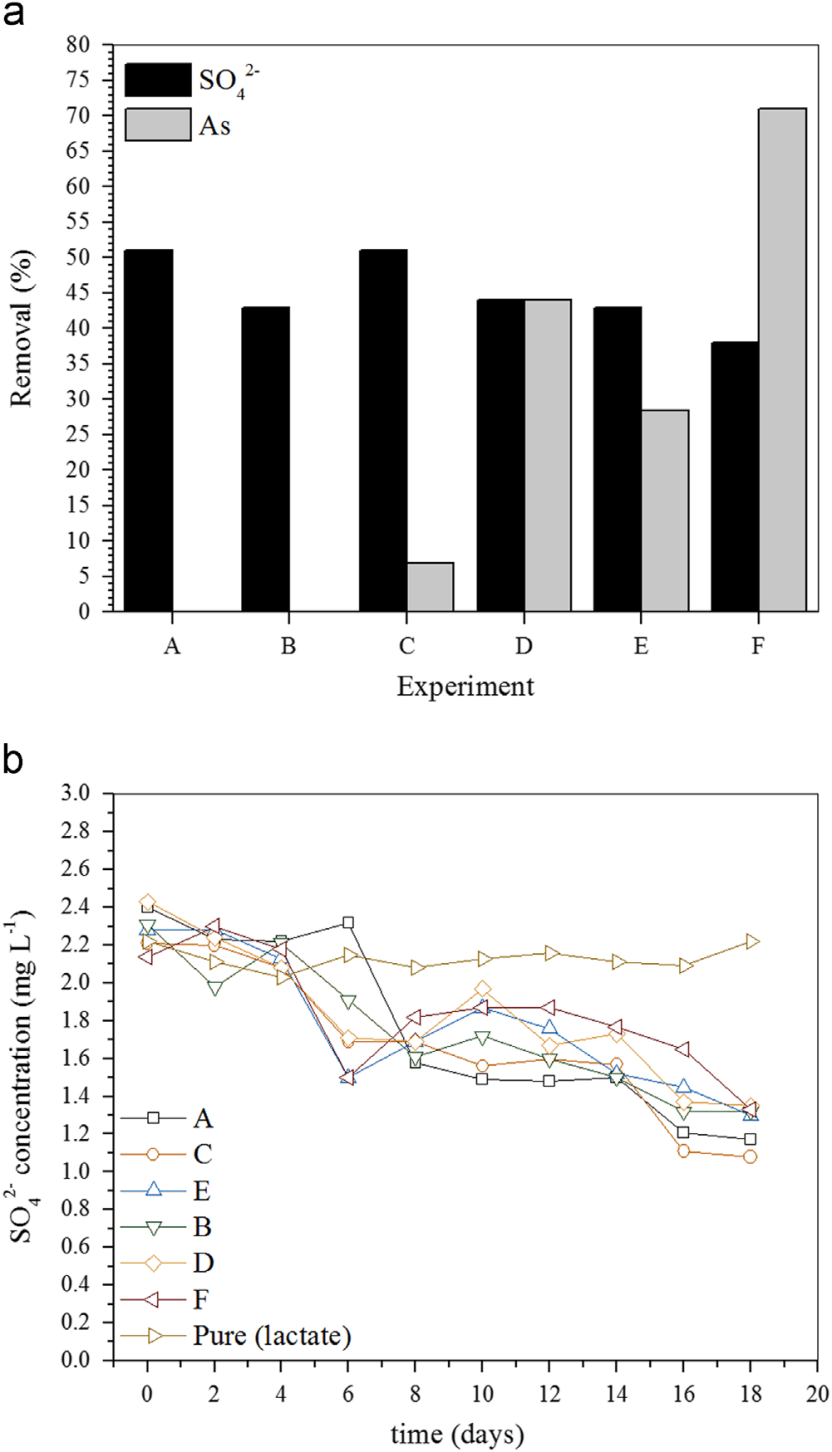
aSulfate and As(III) removal (batch reactors) in acidic medium (pH 5.5) under different experimental conditions. Carbon source, sodium lactate; PCF (B, C, F); As(III), 0 (A, B), 4.0 (C, D) or 8.0 (E, F) mg L^−^^1^. b Monitoring the sulfate removal (batch reactors) in acidic medium (pH 5.5) under different experimental conditions. Carbon source, sodium lactate; PCF (B, C, F); As(III), 0 (A, B), 4.0 (C, D) or 8.0 (E, F) mg L^−^^1^.

**Fig. 2:**
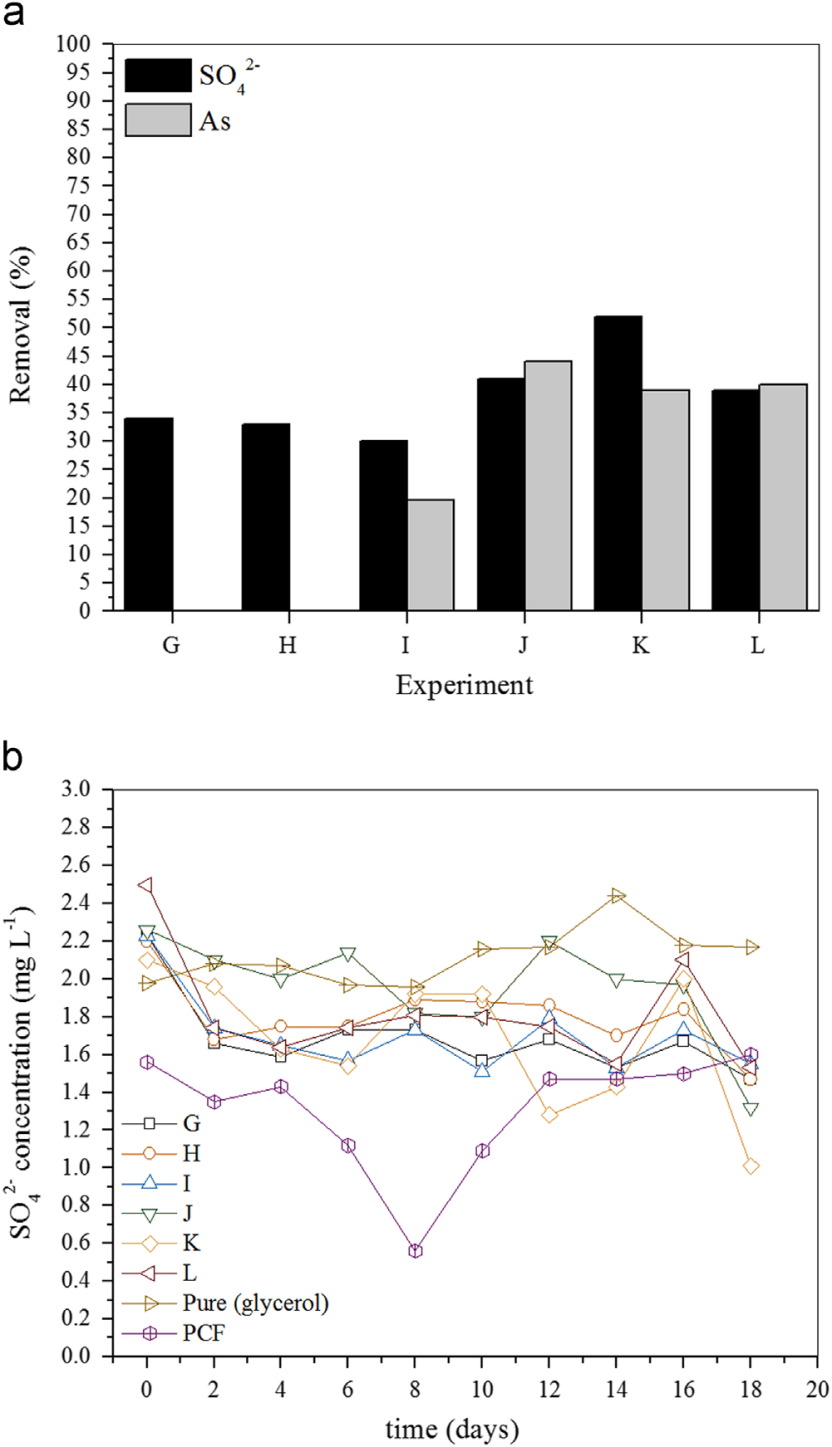
a Sulfate and As(III) removal (batch reactors) in acidic medium (pH 5.5) under different experimental conditions. Carbon source, glycerol; PCF (H, J, L); As(III), 0 (G, H), 4.0 (I, J) or 8.0 (K, L) mg L^−^^1^.b Monitoring the sulfate removal (batch reactors) in acidic medium (pH 5.5) under different experimental conditions. Carbon source, glycerol; PCF (H, J, L); As(III), 0 (G, H), 4.0 (I, J) or 8.0 (K, L) mg L^−^^1^.

**Fig. 3:**
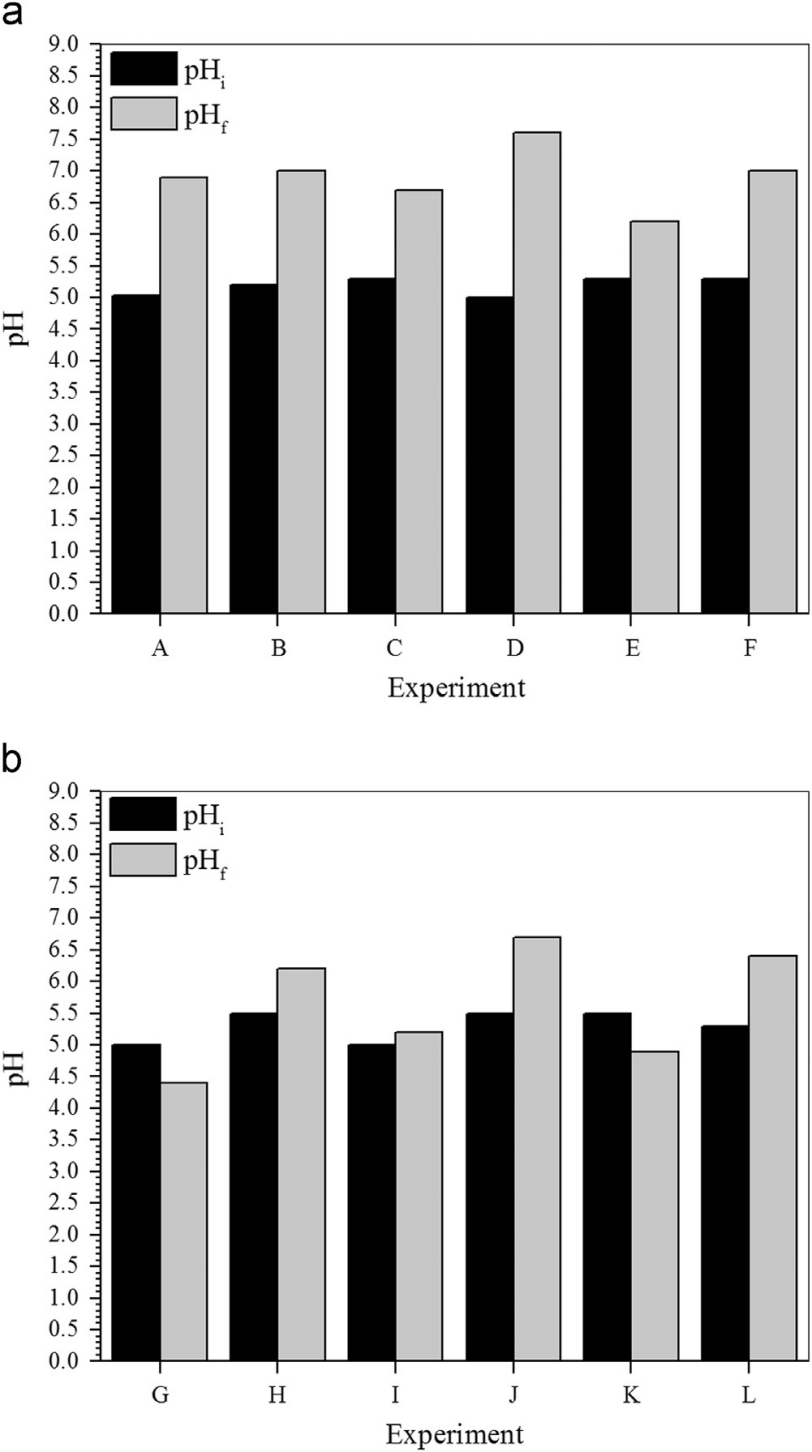
a Changes in pH observed during the microbial growth (batch reactors) under different experimental conditions. Carbon source, sodium lactate; PCF (B, C, F); As(III), 0 (A, B), 4.0 (C, D) or 8.0 (E, F) mg L^−^^1^. b Changes in pH observed during the microbial growth (batch reactors) under different experimental conditions. Carbon source, glycerol; PCF (H, J, L); As(III), 0 (G, H), 4.0 (I, J) or 8.0 (K, L) mg L^−^^1^.

**Fig. 4:**
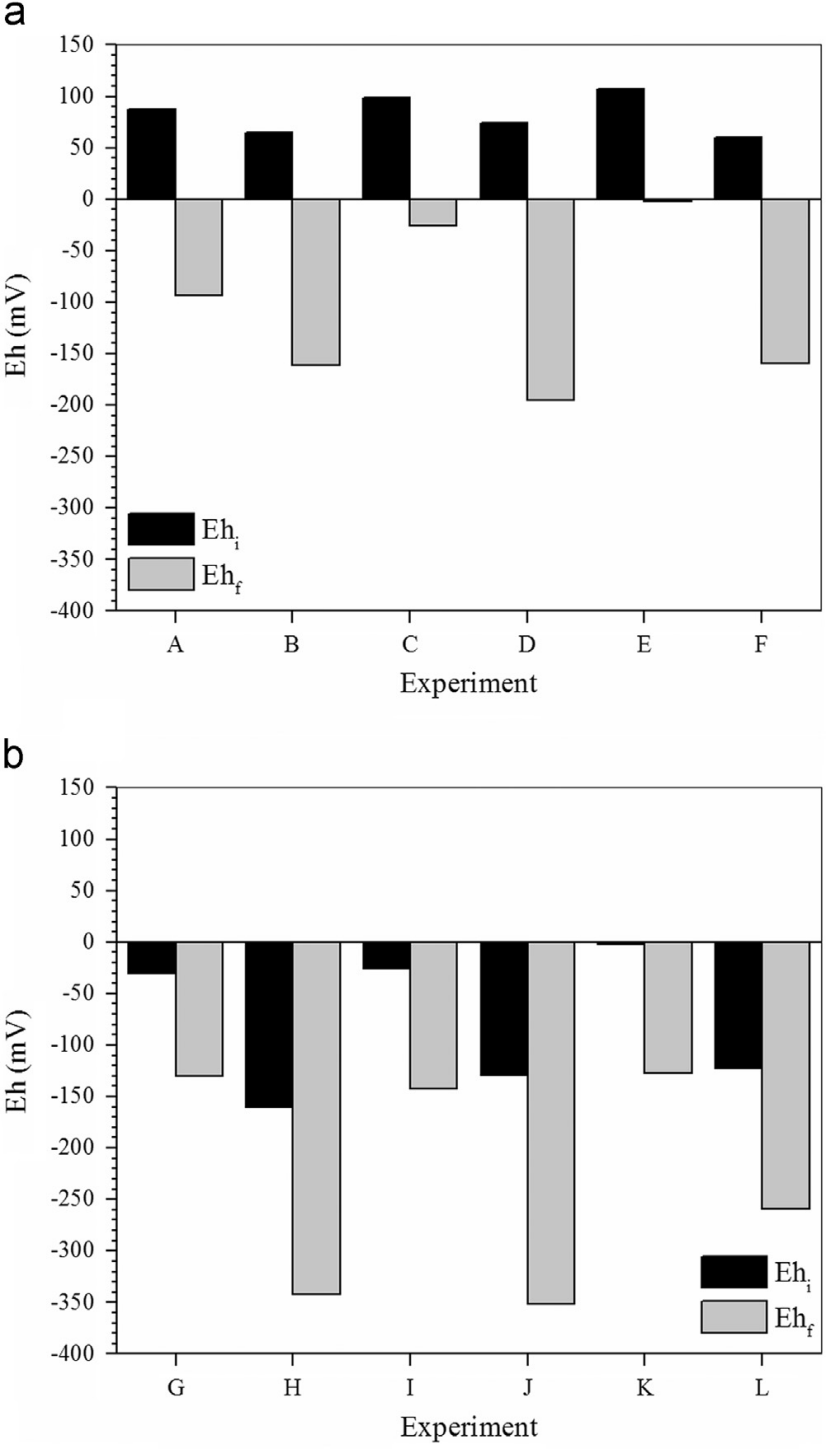
a Changes in Eh (oxidation/reduction potential) observed during microbial growth (pH 5.5) under different experimental conditions. Carbon source, sodium lactate; PCF (B, C, F); As(III), 0 (A, B), 4.0 (C, D) or 8.0 (E, F) mg L^−1^. b Changes in Eh (oxidation/reduction potential) observed during microbial growth (pH 5.5) under different experimental conditions. Carbon source, glycerol; PCF (H, J, L); As(III), 0 (G, H), 4.0 (I, J) or 8.0 (K, L) mg L^−1^.

Data presented in Fig. 1, Fig. 2, Fig. 3, Fig. 4 are referred to experimental conditions summarized in Table 1.

**Table 1:** Batch experiments – visual summary chart.

### 1.2 Semi-continuous experiments

**Fig. 5:**
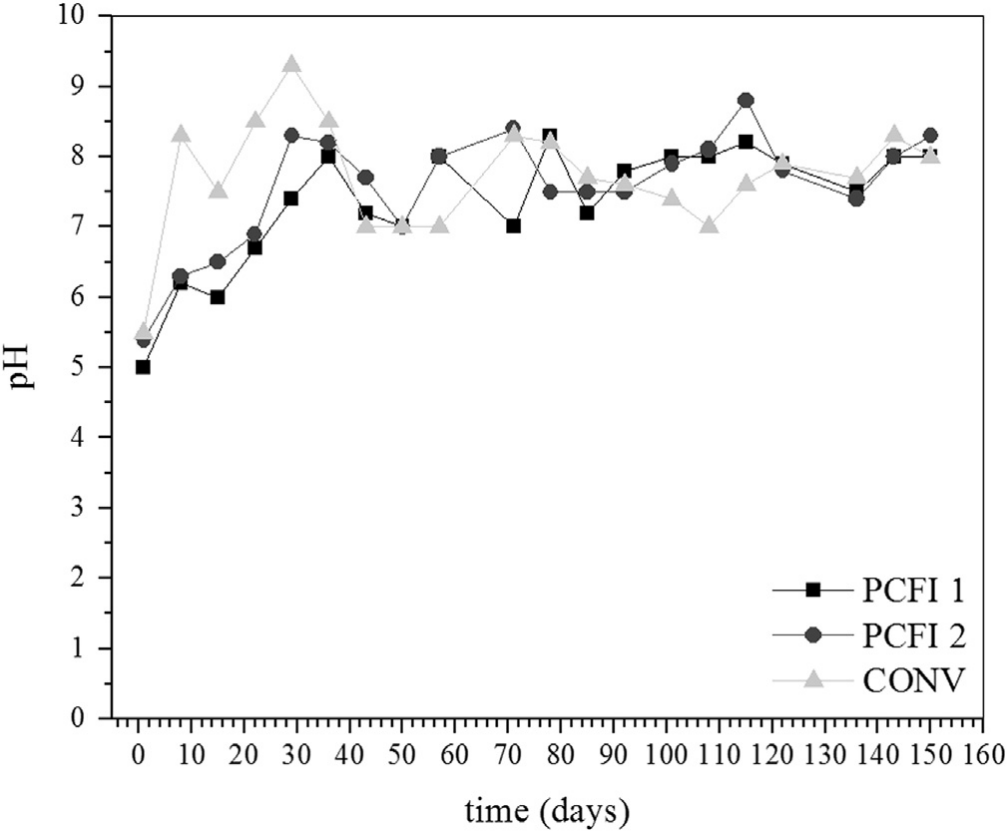
Changes in pH during sulfate and arsenic removal in semi-continuous reactors. PCFI 1: sulfate, immobilized PCF and tap water, PCFI 2: sulfate, immobilized PCF and distilled water and CONV: modified Postgate B liquid medium and distilled water. Initial As(III) concentration of 8.0 mg L^−^^1^, pH 5.0 and 34 °C.

**Fig. 6:**
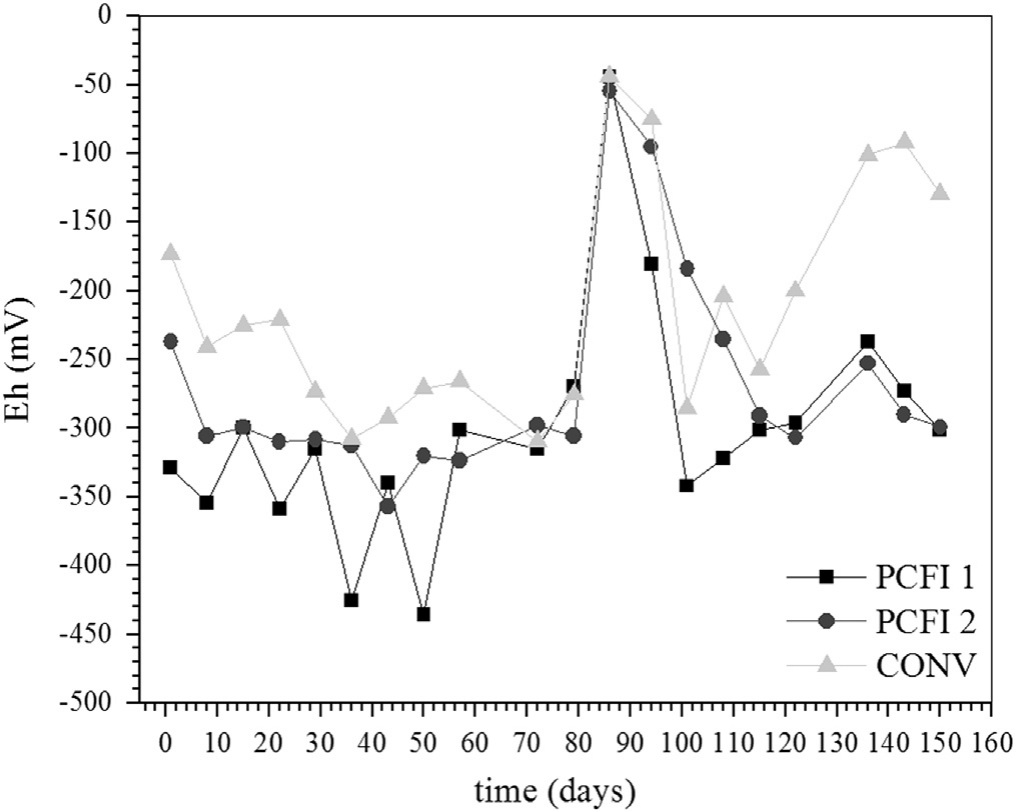
Changes in Eh during sulfate and arsenic removal in semi-continuous reactors. PCFI 1: sulfate, immobilized PCF and tap water, PCFI 2: sulfate, immobilized PCF and distilled water and CONV: modified Postgate B liquid medium and distilled water. Initial As(III) concentration of 8.0 mg L^−1^, pH 5.0 and 34 °C.

Figures 5 and 6 depict some results obtained during semi-continuous experiments. Experiments were carried out in bioreactors operated according conditions described in section 2.2.4.

## 2 Experimental design, materials, and methods

Experimental design is detailed in Matos et al. [1]. All experiments and analyses were replicated twice and data were averaged.

### 2.1 Materials

Analytical grade reagents – Calcium alginate, HNO_3_, HCl, NaOH, KH_2_PO_4_, NH_4_Cl, Na_2_SO_4_, MgSO_4_·7H_2_O, HCl, FeSO_4_, sodium thioglycolate, ascorbic acid, ethylenediaminetetraacetic acid (EDTA), glycerol, sodium lactate - were purchased from different suppliers. A Brazilian poultry plant provided the powdered chicken feathers (PCF) used as solid support material and chemical substrate for microbial growth. Distilled or tap (when mentioned) waters were used for preparing solutions and culture medium.

### 2.2 Methods

#### 2.2.1 Microbial consortium

A microbial culture was obtained after enrichment of some sediment samples, collected from an urban pond, using modified Postgate C liquid medium [1], [2]. Microbial culture was adapted to acidic pH (5–5.5) and to the use of Glycerol as electron donor. Main identified microbial species were *Pantoea agglomerans*, *Enterobacter sp, Citrobacter sp*; *Cupriavidus metallidurans*, *Ralstonia sp* and *Burkholderia cepacia* [1]. Microbial mixed culture was considered as a non-traditional sulfate reducing bacteria (SRB) consortium.

#### 2.2.2 Culture parameters

Microbial growth and process efficiency were indirectly estimated by measuring arsenic and sulfate contents and pH and Eh (mV) changes as well.

Both, batch and semi-continuous experiments were conducted using free and calcium alginate encapsulated microbial cells [1]. Postgate C liquid medium, at pH 5.5 was enriched with sodium lactate, glycerol and PCF as carbon sources. Culture medium pH was acidified to pH 5.5. Microbial tolerance to arsenic (4.0 and 8.0 mg of As(III) L^−^^1^) was accessed.

#### 2.2.3 Batch experiments

Chemical oxygen demand (COD)-to-sulfate ratio used were 2.5 or 3.0, using sodium lactate or glycerol as main soluble carbon sources, respectively. Cultures were incubated in sterilized glass bottles (Postgate B medium, 473 mL, pH 5.0) containing a 5% (w v^−1^) inoculum, PCF (2%) and 4.0 or 8.0 mg L^−^^1^ of As(III). An abiotic control flask was also compared. Flasks were sealed to minimize Oxygen dissolution and incubated at 35 °C for 360 h. 2 mL aliquots were withdrawn for residual sulfate concentration, pH and Eh measurements. Initial and final As(III) concentration were determined.

#### 2.2.4 Semi-continuous experiments

**Fig. 7:**
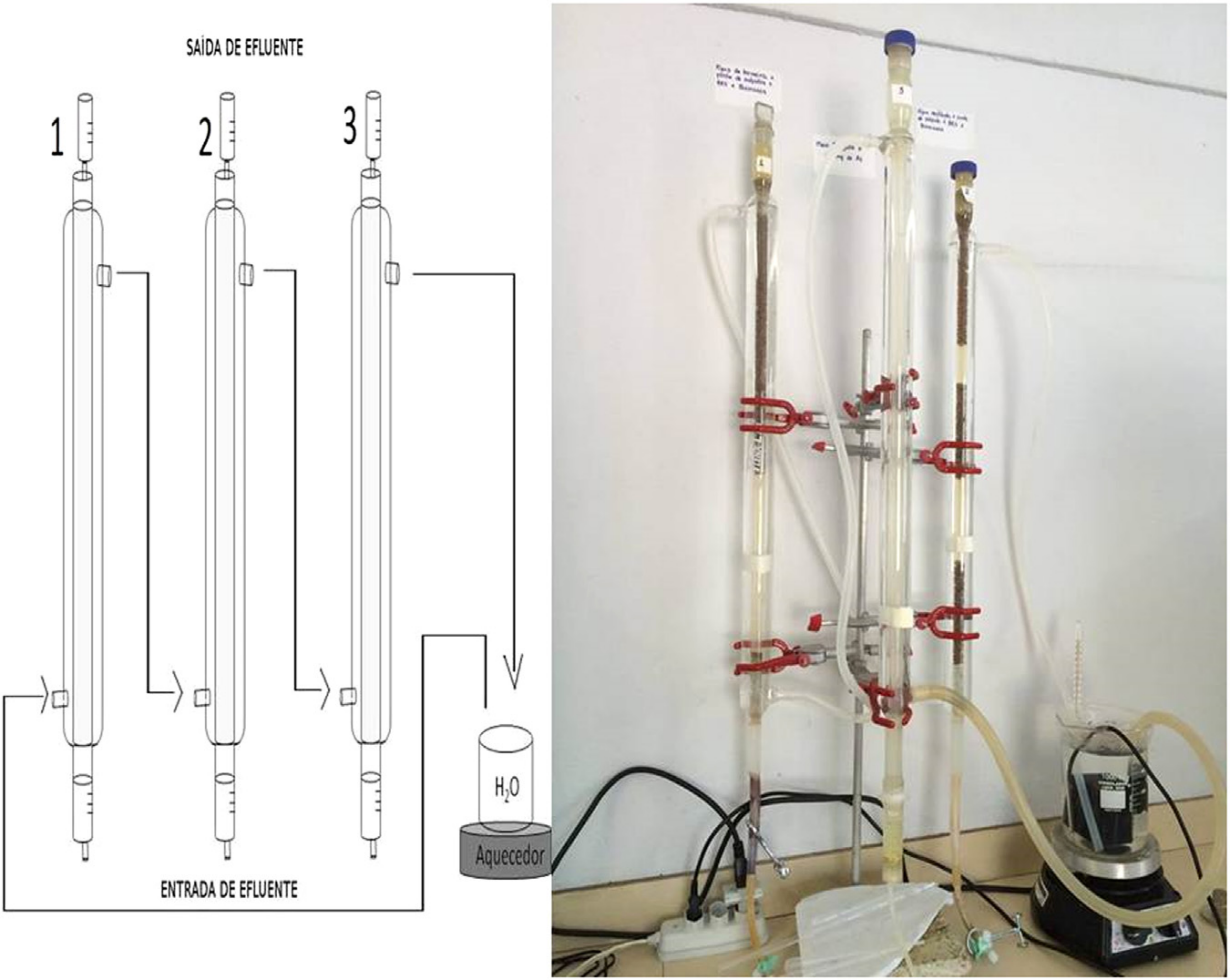
Bioreactors PCFI1, CONV and PCFI2 - Visual aspect.

Four glass bioreactors (Fig. 7) were constructed as described by Matos et al. [1]. Bioreactors were supplied with Postgate C medium and inoculated with microbial consortium adapted to different electron donors and pH values. Sodium lactate and PCF (PCFF, CONV), glycerol and immobilized PCF (PCFI 1, PCFI 2, CONV) and free PCF (PCFF) were tested as carbon sources. Free (PCFF, CONV) or encapsulated (PCFI 1, PCFI 2) microbial cells were inoculated into the systems. Additionally, the suitability of using tap water (PCFI 1) instead of purified water (PCFF, PCFI 2, CONV) was tested. Operating temperature was 34±2 °C and bioreactors were supplied by an up-flow flux. Monitored parameters were pH, oxidation/reduction potential (Eh), residual sulfate and As(III) concentration. Bioreactors were operated during more than 150 days.
