# Supplementary material for: Data set on the bioprecipitation of sulfate and trivalent arsenic by acidophilic non-traditional sulfur reducing bacteria
Source: Data Brief. 2018 Jan 2;17:57–65. doi: 10.1016/j.dib.2017.12.064 (PMC5988216; doi:10.1016/j.dib.2017.12.064)
Supplement: Supplementary file 1 — Supplementary material [file mmc1.docx]

Conflict of interest

Manuscript No.: DIB-D-17-01128
Title: Data set on the bioprecipitation of sulfate and trivalent arsenic by acidophilic non-traditional sulfur reducing bacteria
Journal Title: Data in Brief
Corresponding Author: Dr. Monica Teixeira

Authors’ statement: There is no conflict of interest.
